# Electrospun silk fibroin/fibrin vascular scaffold with superior mechanical properties and biocompatibility for applications in tissue engineering

**DOI:** 10.1038/s41598-024-54638-0

**Published:** 2024-02-16

**Authors:** Lei Yang, Xu Wang, Man Xiong, Xinfang Liu, Sidong Luo, Jinxian Luo, Yeyang Wang

**Affiliations:** 1 Department of Surgical Base, Guangdong Second Provincial General Hospital, Guangzhou, China; 2 Orthopaedic Center, Guangdong Second Provincial General Hospital, Guangzhou, China; 3 Orthopaedic Center, Zhaoqing Central People’s Hospital, Zhaoqing, Guangdong China; 4 The Second School of Clinical Medicine, Southern Medical University, Guangzhou, China; 5 Biomedical College, Guangdong University of Technology, Guangzhou, China; 6 School of Nursing, Guangzhou University of Chinese Medicine, Guangzhou, China; 7 Department of Thyroid and Mammary Surgery, Guangdong Second Provincial General Hospital, Guangzhou, China

**Keywords:** SF/fibrin scaffold, Mechanical strength, Electrospun, Vascular, Tissue engineering, Biological techniques, Cardiology, Engineering, Nanoscience and technology

## Abstract

Electrospun scaffolds play important roles in the fields of regenerative medicine and vascular tissue engineering. The aim of the research described here was to develop a vascular scaffold that mimics the structural and functional properties of natural vascular scaffolding. The mechanical properties of artificial vascular tissue represent a key issue for successful transplantation in small diameter engineering blood vessels. We blended silk fibroin (SF) and fibrin to fabricate a composite scaffold using electrospinning to overcome the shortcomings of fibrin with respect to its mechanical properties. Subsequently, we then carefully investigated the morphological, mechanical properties, hydrophilicity, hemocompatibility, degradation, cytocompatibility and biocompatibility of the SF/fibrin (0:100), SF/fibrin (15:85), SF/fibrin (25:75), and SF/fibrin (35:65) scaffolds. Based on these in vitro results, we implanted SF/fibrin (25:75) vascular scaffold subcutaneously and analyzed its in vivo degradation and histocompatibility. The fiber structure of the SF/fibrin hybrid scaffold was smooth and uniform, and its fiber diameters were relatively small. Compared with the fibrin scaffold, the SF/fibrin scaffold clearly displayed increased mechanical strength, but the hydrophilicity weakened correspondingly. All of the SF/fibrin scaffolds showed excellent blood compatibility and appropriate biodegradation rates. The SF/fibrin (25:75) scaffold increased the proliferation and adhesion of MSCs. The results of animal experiments confirmed that the degradation of the SF/fibrin (25:75) scaffold was faster than that of the SF scaffold and effectively promoted tissue regeneration and cell infiltration. All in all, the SF/fibrin (25:75) electrospun scaffold displayed balanced and controllable biomechanical properties, degradability, and good cell compatibility. Thus, this scaffold proved to be an ideal candidate material for artificial blood vessels.

## Introduction

According to research by the World Health Organization (WHO), including a survey, the mortality rate of cardiovascular diseases (CVDs), greatly exceeds that of other diseases, seriously endangering the health of people worldwide^1^. Thus, in 2019, CVDs were responsible for more than 18 million deaths and 390 million disabilities. According to reports from WHO, the number of deaths caused by CVDs may exceed 23.4 million by 2030^2^. Accordingly, these illnesses impose an enormous economic burden on many countries^3,4^. Contributing to the problem, approximately 0.7% of infants are born with congenital cardiovascular diseases (CCDs). In China, some 200,000 new cases of CCDs are diagnosed annually^5^. Medical professionals widely consider revascularization a satisfactory treatment for severe CVDs, including CCDs, in particular to relieve myocardial ischemia or reduce myocardial damage. The medical decision regarding whether a patient should undergo intervention therapy or coronary artery bypass surgery depends on the severity of these problems. Researchers tend to prefer autologous vessels with good biocompatibility, such as the greater saphenous vein, as a source of blood vessels, but such vessels have drawbacks in terms of being always under-resourced and displaying anatomical variation^6^. Therefore, the development of suitable artificial vascular scaffolds is desirable to facilitate the treatment of these diseases.

Many studies over more than 70 years have confirmed the success of synthetic vascular grafts of large diameter vessels (Ø > 6 mm) in clinical practice^7^. Regrettably, ideal results have proved elusive when the material has been applied to small-diameter vessels (Ø < 6 mm), an outcome that has been attributed to its relatively low patency rate and susceptibility to calcification, thrombosis and neointimal hyperplasia^8^. These drawbacks have hindered the development of vascular grafts, but approaches involving tissue engineering have the potential to overcome them. Thus, there is a need for small-diameter vascular scaffolds with mechanical and biological characteristics similar to those of autologous vessels that function stably under hemodynamic conditions. The development of such scaffolds has the potential to initiate a profound revolution in the treatment of CVDs.

Currently, many researchers are using a wide range of synthetic polymer materials in particular Silk fibroin (SF), polycaprolactone, polyurethane and poly(l-lactide-co-ε-caprolactone), in the effort to develop small diameter tissue engineered vascular grafts^9,10^. The advantages of these materials include superior biocompatibility, desirable mechanical properties and cell affinity. While the disadvantages of insufficiency in degradability and biological stability, typically hydrophobic, which are unfavorable to cell proliferation and adhesion. Consequently, investigators tend to focus on combining polymers to compensate for individual shortcomings, often blending native materials and polymer materials for the ideal composite material. For example, Ikram et al.^11^ employed fibrin as an adjuvant to fabricate mechanically stable SF/fibrin biocomposites using a simple and scalable technology, and concluded that these materials are suitable for cardiovascular tissue engineering.

SF is a natural polymer, usually derived from mulberry Bombyx mori cocoons. Furthermore, many researchers have confirmed that SF is an ideal biological scaffold material because of its excellent mechanical properties, low immune reaction, superior biocompatibility, and presentation of various polymer modification sites and bioactive sites^12^. Whereas, insufficient processability and slow degradation have limited the application of SF in vascular tissue engineering. Fibrin has attracted the attention of researchers in recent years. Compared with other natural biomaterials, it can be obtained at relatively little expense from patients’ blood and its preparation is relatively inexpensive and completely autologous. In addition, its bioactive matrix is advantageous in terms of promoting biochemical molecules and cell-delivery systems^13^. Though fibrin also has some disadvantages, including slow degradation and insufficient mechanical properties, many researchers have incorporated it into composite vascular materials for their application^14^.

For the present study, we designed and developed four different types of SF/fibrin vascular scaffolds by electrospinning. The composite scaffolds showed greatly improved their mechanical properties and biocompatibility. We comprehensively investigated the morphology, structure, hydrophilicity, biomechanical properties, hemocompatibility, and degradation in vitro of the various SF/fibrin vascular materials fabricated through electrospinning. We also used a cytocompatibility test and characterization of in vivo implantation to evaluate the biological properties of the scaffolds. Based on the results, we hypothesized that the electrospun SF/fibrin vascular scaffold with the optimum mass ratio display mechanical strength comparable to that of native blood vessels. We expect that the properties of these scaffolds will make them useful in the engineering of small-diameter tissue-engineered vascular grafts.

## Materials and methods

### Materials

Fibrin was provided by Thermo Fisher (Wuhan, China). Bombyx mori silk cocoons were obtained from Chuangseed Biomaterials Company (Guangzhou, China). 98% formic acid and Dulbecco's Modified Eagle Medium (DMEM) was obtained from Dingguo Biotechnology Co., Ltd (Zhengzhou, China). Cell Counting kit-8, Masson staining kit, CD68, hematoxylin eosin staining kit and Live Cell Staining Kit were acquired from APExBIO Technology LLC (Shanghai, China). Fetal bovine serum, actin antibody, DAPI, Triton-X100, PBS and 4%polyformaldehyde solutions were purchased from Weigo Technology Co., Ltd (Guangzhou, China).

### Fabrication of SF/fibrin vascular materials

The members of our research team have abundant experience and achieved satisfactory results in work similar to the preparation of SF/fibrin vascular scaffolds by electrospinning^15,16^. The first step was the extraction of the SF. Bombyx mori silk cocoons were prepared into smaller pieces and soaked them in ethanol for 48 h. Then, we put them in Na_2_CO_3_ aqueous (0.5% w/v) solution and boiled them for an hour with the material to liquor ratio of 1:100. This operation was carried out at a temperature of 100 °C. Subsequently, the as-degummed silk fibroin was washed with distilled water multiple times, and then dried in an oven for 24 h, and dissolved it in CaCl_2_–CH_3_CH_2_OH–H_2_O (mole ratio = 1:2:8) at 76 ± 2 °C for 5 min. After that, the SF solution was dialyzed against distilled water for about 72 h. The SF solutions were lyophilized before they were storaged^17,18^. Briefly, SF/fibrin with mass ratios of 0:100, 15:85, 25:75 and 35:65 were dissolved into formic acid solvent to obtain a fixed mixing concentration (10 wt%), respectively. They were then placed on a shaking table and shaken for 12 h to prepare some homogeneous electrospinning solutions. The parameter values of the electrospinning instrument (FM1012, Dalian, China) were as follows: curing distance of 10 cm, volume flow rate of 1 mL/h and a spinning voltage of 20 kV. Various SF/fibrin nanofibers were obtained after the polymer solution was cured by high voltage electricity. Eventually, the prepared scaffold was dried and ventilated to eliminate the residual solvent.

### Exploration of SF/fibrin scaffolds using SEM

Scanning electron microscopy (SEM, Hitachi SU8100, Japan) was used to analyze the morphological changes of SF/fibrin vascular scaffolds. All of the samples were prepared with the same area size, sputtered with gold for half a minute, and then observed under SEM with an acceleration voltage of 10kV. Our method for evaluating the mean fiber diameter distribution of the electrospun materials used Image J software. Fifty nanofibers were randomly chosen, and their mean diameters were analyzed, and then the corresponding images were drawn.

### Contact angle examination

The hydrophobicity of different SF/fibrin vascular scaffolds was measured by contact angle equipment (Fangrui, China). Specifically, four different samples were prepared into a 1 cm × 1 cm square and placed them on the stage. Afterwards, we placed the different samples on the stage and sequentially dropped syringes with distilled water onto the surface of the sample. A camera collected the images 5 s after the droplets were deposited. The contact angle was calculated by computer software. In the meantime, these experimental samples were checked three times and the mean value was achieved (n = 3).

### Investigation of mechanical test in vitro

#### Burst strength

To obtain the ideal burst strength value, four of the tested samples were completely hydrated in PBS for 20 min (n = 3). Then, we fixed the samples on the equipment with 3–0 silk sutures and blocked the other ends. We steadily injected PBS solution into the samples until the vascular scaffolds ruptured. We used the CPT2500 USB installation to record the maximum pressure data before the failure of the test samples and took it as the burst strength value.

#### Suture strength

The suture strength of the vascular scaffolds met the standards of the China Association of Medical Devices Industry (CAMDI) to fully guarantees its safety and effectiveness. We prepared different vascular materials with a length of about 2.0 cm, and fixed them at one end of the instrument with 6–0 polypropylene sutures. The other end was located in the grip, and the looseness of the equipment before their operation was checked. Next, we operated with an invariable elongation rate until the failure of the samples and retained the maximum load of the samples prior to tearing off. These values were considered as the size of suture strength (n = 3).

#### Tensile properties

A universal material testing machine (WDW-10, Yanrun, China) served to examine the mechanical properties of four different SF/fibrin scaffolds in axial directions. The as-prepared materials were sequentially split into 2.0 cm × 1.0 cm sizes, and the electrospun materials of each sample were evaluated. To assessment the tensile strength of the materials, we adjusted the appropriate parameter values tested in the device. The mechanical properties of different samples were measured by testing equipment. Each test samples were checked three times, and the mean value was analyzed and obtained.

### Assessment of blood-materials compatibility

#### Hemolysis assay

We conducted a hemolysis experiment to evaluate the biological safety of the vascular scaffolds with the blood cells. Fresh anticoagulated blood from Sprague Dawley (SD) rats was thoroughly mixed with normal saline, and then 2% of the mixture was prepared. The diluted blood solution (0.2 mL) was placed in the sterilized SF/fibrin samples respectively. The mixture was placed in a container at 37 °C and incubated for half an hour. Then, the various sample solutions were then centrifuged for 10 min at 1500 r/min. The supernatant was retained in a 96-well plate, and examined by the absorbance value of the microplate analyzer (Microplate, China) at 540 nm. These samples were tested three times to obtain the mean value. The components of the positive group were diluted blood (0.2 mL) and distilled water (3.8 mL). While, diluted blood (0.2 mL) and normal saline (3.8mL) were combined with the negative group. The analysis formula of hemolysis rate (HR) was as follows:

$${\text{HR}}\;{(}\% {)} = \frac{{{\text{Dt}} - {\text{Dnc}}}}{{{\text{Dpc}} - {\text{Dpc}}}} \times {1}00\%$$

where Dt indicated the absorbance of the test scaffold, Dnc referred to the absorbance of the negative control, and Dpc showed the absorbance of the positive control.

#### Platelet adhesion test

To obtain fresh anticoagulant whole blood, we mixed fresh blood from SD rats with sodium citrate. Moreover, we placed it in an instrument and centrifuged it at 1000 rpm for 15 min to obtain the supernatant consisting of platelet-rich plasma (PRP). Then, various samples of nanomaterials were then sterilized by ultraviolet radiation for about 2 h, and safely placed in 24-well plates. In addition, we repeatedly used PBS for rinsing to obtain clean electrospun materials. The prepared PRP was soaked in various vascular materials and incubated in a 37 °C water baths for 1 h. The PRP was washed in PBS, and then immobilized in 2.5% glutaraldehyde, and the gradient alcohol (100%, 90%, 80%, 70%, 60% and 50%) was dehydrated. After the test samples were dried, we observed the platelet adhesion morphology of the samples by a SEM and collected some images of them.

#### Co-incubation of red blood cells and materials assay

We centrifuged the whole blood, and then removed the supernatant to obtain the compressed red blood cells. After that, we added an appropriate amount of PBS buffer to wash the compressed red twice. The different sterilized vascular scaffolds were evenly distributed on a 24-well plate, and red blood cell solution (700μL) was successfully added to each well. We used a pipette to repeatedly blow on and aspirate the scaffolds repeatedly so as to mix them evenly with the sample. The samples were then incubated for 3 h in a water bath at 37 °C. After they had dried satisfactorily, we analyzed the microscopic morphology of red blood cells on the vascular scaffold under SEM and photos were collected and took photographs.

#### Plasma recalcification time experiment

To analyze the time of blood coagulation of the vascular materials in the calcium-free anticoagulant plasma after another addition of calcium ions, we created platelet-poor plasma (PPP) by centrifuging fresh whole blood at 1800 r/minute for 30 min. Four different samples were taken 4 cm × 4 cm and kept in each sterile tubes. Then, 200μL PPP and 200μL CaCl_2_ solutions were added to each tube, respectively. Hereon, the control group consisted of samples with TCP and without any vascular scaffold material. We observed the earliest time of fibrin formation and recorded then associated data. Each group of samples was evaluated three times, and the mean value was calculated.

#### Coagulation function trial

To study the anticoagulant properties of the vascular material, we used APTT and TT method to evaluate them. To do so, we formed the sterilized vascular scaffolds into a disc shape and stored them in a tube. Then, plasma (1 mL) from rats was added and incubated the tubes for 1 h. We used coagulation equipment (SYSMEX CA-7000, Japan) to analyze the time. A non-vascular substance group served as the control group. We performed the result in triplicate to obtain the experimental data.

### Evaluation of the biodegradation of electrospun materials in vitro

To conduct the in vitro degradation experiment on the various SF/fibrin composite scaffolds, we cut the as-obtained samples into 1 cm × 1 cm pieces. Subsequently, the tested samples were immersed in PBS (pH 7.4) and lipase solution (pH 7.4, 1 mg/mL) successively, and incubated them at 37 °C. After incubation at different specific time points respectively, we weighed the different samples, and then carefully evaluated the mass loss rate using the degradation calculation method (n = 3). The formula of degradation mass loss was one often used in our previous work^15^. We added the PBS solution and supplemented it with the lipase solution once a week.

### Analysis of cell-materials compatibility

#### Cell grow behavior with material extracted

To investigate the compatibility of the material extract with living cells together, we co-cultured rabbit adipose-derived mesenchymal stem cells (RADMSCs, Guangdong Medical Laboratory Animal Center, China) with various scaffold materials. We placed the cells on the culture plates and cultured them at a density of 1 × 10^4^/mL, and then the incubated them at 37 °C in 5% CO_2_ for 2, 4, and 6 days, respectively. Here, samples of cells without scaffold materials served as the control group. We regularly updated the culture medium every 2 days. We then analyzed the cells using a microscope and obtained images of them.

#### Cell proliferation on scaffold materials

An CCK-8 kit served to analyze quantitatively the proliferation of the cells at predetermined time points of culture, respectively. To begin with, four different scaffold materials were prepared into appropriately sized circular discs and co-incubated with RADMSCs, and then placed in 96-well plates for 1, 3, and 7 days. Samples of cells without scaffold materials were used as the control group. After that, we subsequentially added DMEM and CCK-8 overnight in the dark, rinsed repeatedly the samples three times with sterile PBS, and incubated them at 37 °C for 4 h. Lastly, we evaluated the absorbance value at various time points at a wavelength of 540 nm using a fully automatic multifunctional enzyme analyzer equipment (Berthold, Germany).

#### Live cell staining test

After sterilizing the prepared samples with ultraviolet rays, we placed them on 48-well plates. Then, DMEM and 1 × 10^4^ cells were added to per well orderly, and incubated in a CO_2_ incubator at 37 °C. We changed the medium every 2 days. Furthermore, we also added calcein-AM solution at the ideal ration to the samples in accordance with the instructions provided with the cell-staining kit. We drew off the waste liquid and rinsed the cells with PBS to remove impurities. Working in the dark, we added the prepared staining solution evenly to each well plate (100 μL), wrapped the plates in aluminum foil, and incubated them at 37 °C for 30 min, repeatedly rinsing them with PBS. We observed the samples with an inverted fluorescence microscope (DMIL, China) and collected images of them at various time points.

#### Immunofluorescence test

We cut the SF/fibrin electrospun vascular scaffolds into 1.0 cm × 1.0 cm pieces/samples. The test samples were sterilized for 2 h. We washed them repeatedly with PBS, and soaked them. We also co-incubated 20μL of 1 × 10^4^ RADMSCs with various sample materials at the specific time point. After 4 and 7 days of culturing, we washed the samples with PBS for three times, fixed them with a 4% polyoxymethylene solution. Simultaneously, we used 0.2% Triton-X100 to permeate them for 10 min, and obstructed them with 1% bovine serum albumin at 4 °C. Actin antibodies were added to each well in the dark, and the wells were placed in an incubator for 1h at 37 °C, and then stained with DAPI for half an hour in the dark room temperature. We then used a fluorescence microscope (DMIL, China) to observe all the tested samples and collect these images of them. We evaluated the number of cells on each different sample from random fields of view (FOV) with Image J software. Each test sample was evaluated three times.

### Exploration of the properties of electrospun scaffold in vivo

#### Animal experiments

We conducted the subcutaneous implantation assay in vivo was followed in accordance with the Animal Care and Experiment Committee guidelines. Thus, the Animal Ethics Committee of Guangdong Second Provincial General Hospital (Guangdong, China) approved the experimental protocol. We selected 12 SD rats (7 weeks old, male, average weight 100g) from Guangdong Second Provincial General Hospital. We grouped the rats arbitrarily into three groups and implanted the treatment groups with electrospun scaffolds in vivo. We grouped the rats arbitrarily into three groups and implanted the treatment groups with electrospun scaffolds in vivo. Thus, the control group (n = 4) received no scaffolds, one treatment group received SF vascular scaffolds (n = 4), and the other treatment group received SF/fibrin (25:75) vascular scaffolds (n = 4). The prepared samples of the appropriate size were irradiated with ultraviolet light, soaked in ethanol and rinsed with PBS, respectively. We then anesthetized the rats with inhalation of isoflurane and firmly fixed them on an operating table. After removing, we removed the hair in the operation region with an electric razor and sterilizing the skin with 75% medical alcohol, a surgical incision about 2 cm long was cut into the dorsal area of the rats, and the subcutaneous pockets were gently separated with vascular forceps. During the operation, we were careful to protect the important nerves and blood vessels and maintain sterile conditions. We implanted the different sterilized samples into the pockets, and then closed the subcutaneous tissue. The rats continued to drink water and eat food regularly after the operations and were sacrificed. We then removed the electrospun scaffolds along with the surrounding tissues for analysis and investigation.

#### Weightlessness of electrospun scaffold

To investigate the degradability of different vascular scaffolds in vivo, we took out these scaffold materials from the rats in vivo at specific time points, and air-dried them to remove the excess water. We then the weighted the electrospun scaffolds were on an electronic balance. The formula for calculating the weight loss rate was as follows.

$${\text{Weight loss rate}}\;(\%) = \frac{{{\text{W}}1 - {\text{W}}2}}{{{\text{W}}1}} \times 100\%$$

W_1_ showed the dehydrated of the vascular scaffold before implantation, and W_2_ showed the dehydrated of the test sample after implantation.

#### Tissue staining analysis

The SD rats were sacrificed by overdose of anesthetics after 1 and 2 weeks, respectively. We note that one rat died unexpectedly and for unknown reasons during the animal experiment, so the survival rate was 91%. We then collected the implanted scaffolds along with the surrounding tissues, fixed them in 4% polyformaldehyde solutions and ethanol at various concentrations for dehydration, and embedded them in fixed paraffin. We prepared cross-sectional slices of the test samples (thickness 5μm) by microtome (SM2500, Leica, Nussloch, Germany). We stained these slices with hematoxylin–eosin (H&E), Masson's trichrome staining and CD68 observed the samples, collected images of them using an inverted fluorescence microscope (DMIL, China).

### Statistical analysis

The data were indicated by the mean ± standard deviation (SD). We performed these calculations for each experimental group at least three times. We used GraphPad Prism 7 and OriginPro2018 software for statistical analysis and the one-way ANOVA method was applied to compare the mean values within each group. Values of *p* < 0.05 indicated a significant difference.

### Ethical approval

All animal experiments in the manuscript were approved by the ARRIVE guidelines.

## Results

### Preparation and morphology of the SF/fibrin vascular scaffolds

**Figure 1:**
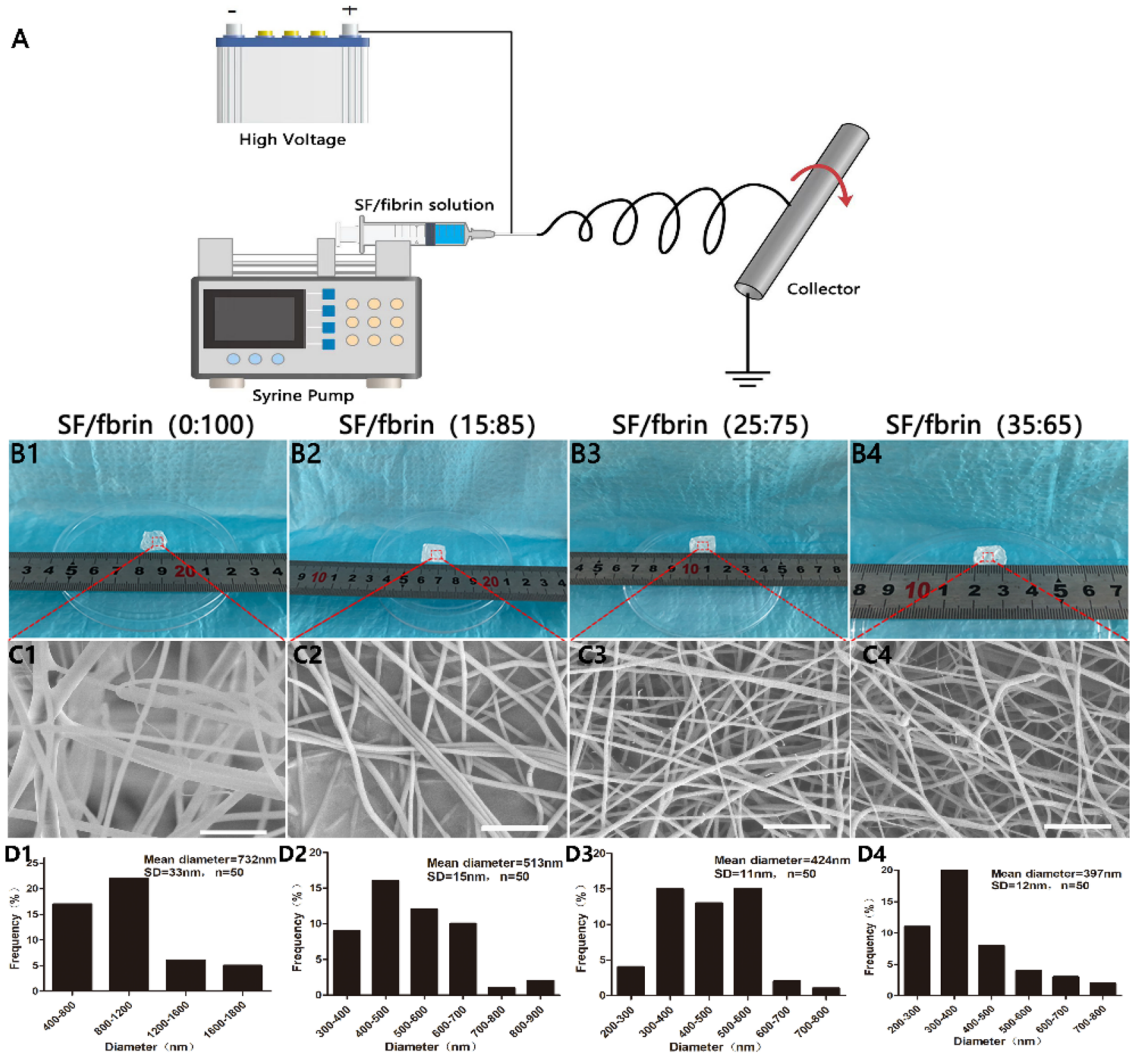
Fabrication and characterization of the electrospun vascular scaffolds. Schematic diagram of the electrospun SF/fibrin hybrid scaffolds (**A**). The macroscopic appearance of SF/fibrin scaffolds with different mass ratios (**B1–B4**). SEM images of four electrospun scaffolds (**C1–C4**). Scale bars: 10 μm. Fiber diameter distribution of different SF/fibrin scaffolds by ImageJ software (**D1–D4**) (n = 3).

Using SF and fibrin as raw materials, we designed and developed a series of SF/fibrin vascular scaffolds with different mass ratios using an electrospinning system (Fig. 1A). The results indicated that we were successful fabricating four different SF/fibrin electrospun films measuring 1.2 cm (length) × 1.2 cm (width) (Fig. 1B1–B4). To further explore the internal microscopic morphology of the vascular scaffolds, we found under the SEM that the nanofiber structure of four different SF/fibrin scaffolds were smooth, without obvious bead-like defects, and that the fibers were randomly distributed (Fig. 1C1–C4). What’s more, we randomly chose 50 nanofibers under the SEM and evaluated their fiber diameters using ImageJ software. The analysis showed that the mean fiber diameters of the SF/fibrin (0:100), SF/fibrin (15:85), SF/fibrin (25:75) and SF/fibrin (35:65) were 732 ± 33 nm,513 ± 15 nm,424 ± 11 nm, and 397 ± 12 nm, respectively (Fig. 1D1–D4).

### Hydrophilicity and mechanical characterization of the SF/fibrin scaffolds

**Figure 2:**
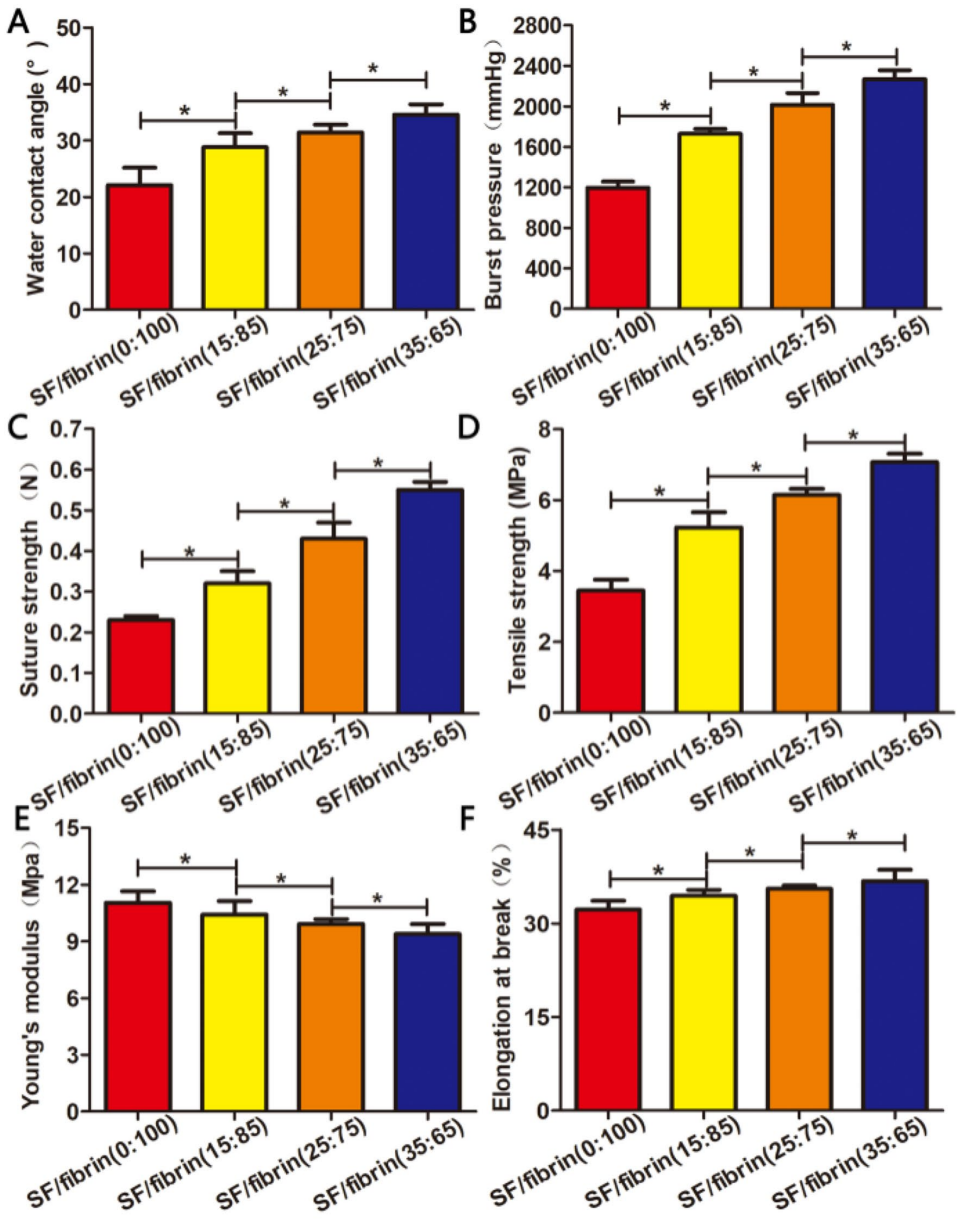
Analysis of the hydrophilicity and mechanical properties of the four SF/fibrin vascular scaffolds. Water contact angel of the four electrospun scaffolds (**A**). The burst pressure (**B**), suture strength (**C**), tensile strength (**D**), Young's modulus (**E**) and elongation at break (**F**) of the four SF/fibrin scaffolds (n = 3). *P < 0.05.

To analyze the hydrophilic properties of the samples, we evaluated their water contact angles. The results from the water contact angle experiment showed that the contact angle of the SF/fibrin (0:100) electrospun scaffold was about 22.1°, and that the contact angle data of the four scaffolds increased as the SF content increased. The water contact angel of the SF/fibrin (35:65) vascular scaffold was 34.6° confirmed that the increased in the SF/fibrin mass ratio affected the contact angle value (Fig. 2A).

For a tissue engineering vascular scaffold, the key measurement index is burst pressure. When the SF content in the SF/fibrin composite scaffolds increased from 15 to 35%, the burst pressure increased from 1729 ± 48 mmHg to 2267 ± 97 mmHg, respectively. Moreover, the results showed that the burst pressure of SF/fibrin (0:100), SF/fibrin (15:85), SF/fibrin (25:75) and SF/fibrin (35:75) scaffolds were significant differences (Fig. 2B).

Suture strength of the vascular scaffold is an important evaluation index with respect to the success or failure of artificial vascular scaffold transplantation. We found that the suture strength data of four SF/fibrin electrospun scaffolds increased significantly as the SF content increased. Interestingly, the suture strength of the SF/fibrin (15:85), SF/fibrin (25:75) and SF/fibrin (35:65) scaffolds were comparable to those of native arteries. Meanwhile, the suture strength values of the four SF/fibrin scaffolds were statistically significant (Fig. 2C).

The ideal electrospun scaffold material would have excellent mechanical strength and the stability to maintain a stable structure and resist bloodstream movement. Accordingly, we were attentive to the mechanical properties of our scaffold. The results shown in Fig. 2D–F indicated that compared with the pure fibrin scaffold, the tensile strength and elongation at break of the other SF/fibrin scaffolds were significantly improved, but the Young's modulus was reduced. More specifically, the mechanical strength of the four SF/fibrin scaffolds increased significantly as the SF content increased.

### Hemocompatibility of the SF/fibrin vascular scaffolds

**Figure 3:**
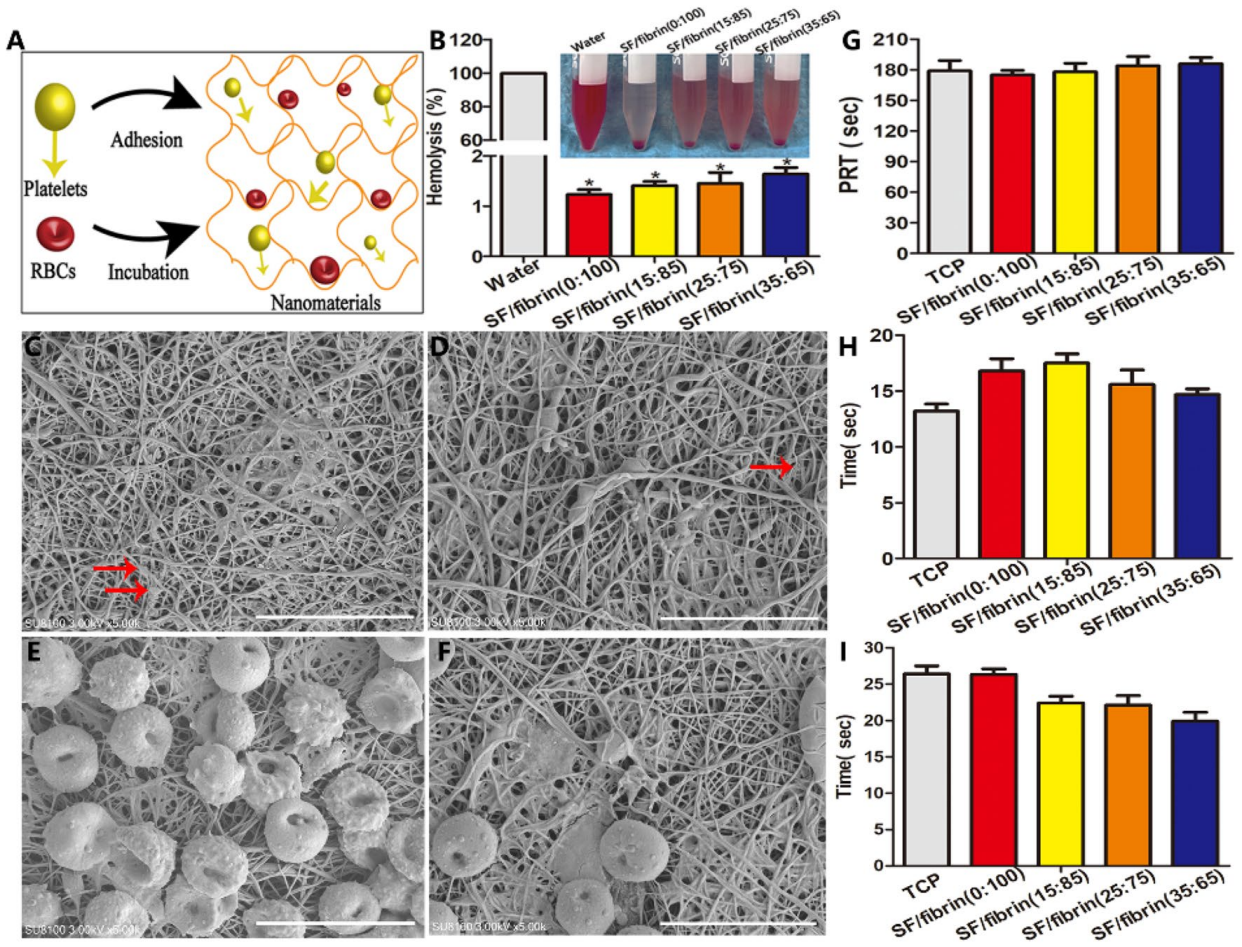
Schematic diagram of platelet adhesion and red blood cell incubation on the vascular scaffolds (**A**). Hemolysis rate of the four different scaffolds material (**B**). Images of platelet adhesion in SF/fibrin (**C**) and fibrin (**D**) samples under SEM. The red arrows referred to platelets adhered under the SEM. SEM photos of the red blood cells incubated with fibrin (**E**) and SF/fibrin (**F**) vascular scaffold for 3 h. Scale bars: 10 μm. The Plasma recalcification time (**G**), TT (**H**) and APTT (**I**) were evaluated (n = 3). *P < 0.05.

The schematic diagram of platelet adhesion and red blood cell incubation on scaffold materials were showed in Fig. 3A. Compared with the control group, hemolysis was infrequent on the four vascular scaffold materials. Furthermore, the hemolysis value of four different SF/fibrin scaffolds were lower than 2%, and less than the American Society for Testing and Materials standard value of 5% (Fig. 3B). To study the behavior of the platelets on the different vascular scaffolds, we analyzed their morphology by SEM. The SEM images showed that a small number of sporadic platelets adhered to the surface of the vascular materials, but no significant aggregation or deformation occurred (Fig. 3C, D). These results indicated that the vascular scaffold had excellent anticoagulant properties. Interestingly, we observed no significant deformation of erythrocyte morphology on fibrin or SF/fibrin electrospun membranes, and this result further indicated that these vascular materials have good hydrophilic properties (Fig. 3E, F).

The plasma recalcification time (PRT) is an experimental indicator of defects in the endogenous coagulation system. The PRT values of SF/fibrin (0:100), SF/fibrin (15:85), SF/fibrin (25:75) and SF/fibrin (35:65) electrospun vascular scaffolds were 175 s, 178 s, 184 s and 186 s, respectively. Meanwhile, there was no statistical difference among the groups, and then the PRT value decreased slightly with the increase in SF content (Fig. 3G). Moreover, the results showed that TT values of the four different SF/fibrin electrospun scaffolds were all in normal safety range (15–18 s) (Fig. 3H). Surprisingly, as the SF content of the SF/fibrin scaffolds increased, the value of APTT did not change significantly (Fig. 3I).

### The biodegradability and cell growth with scaffold extract

**Figure 4:**
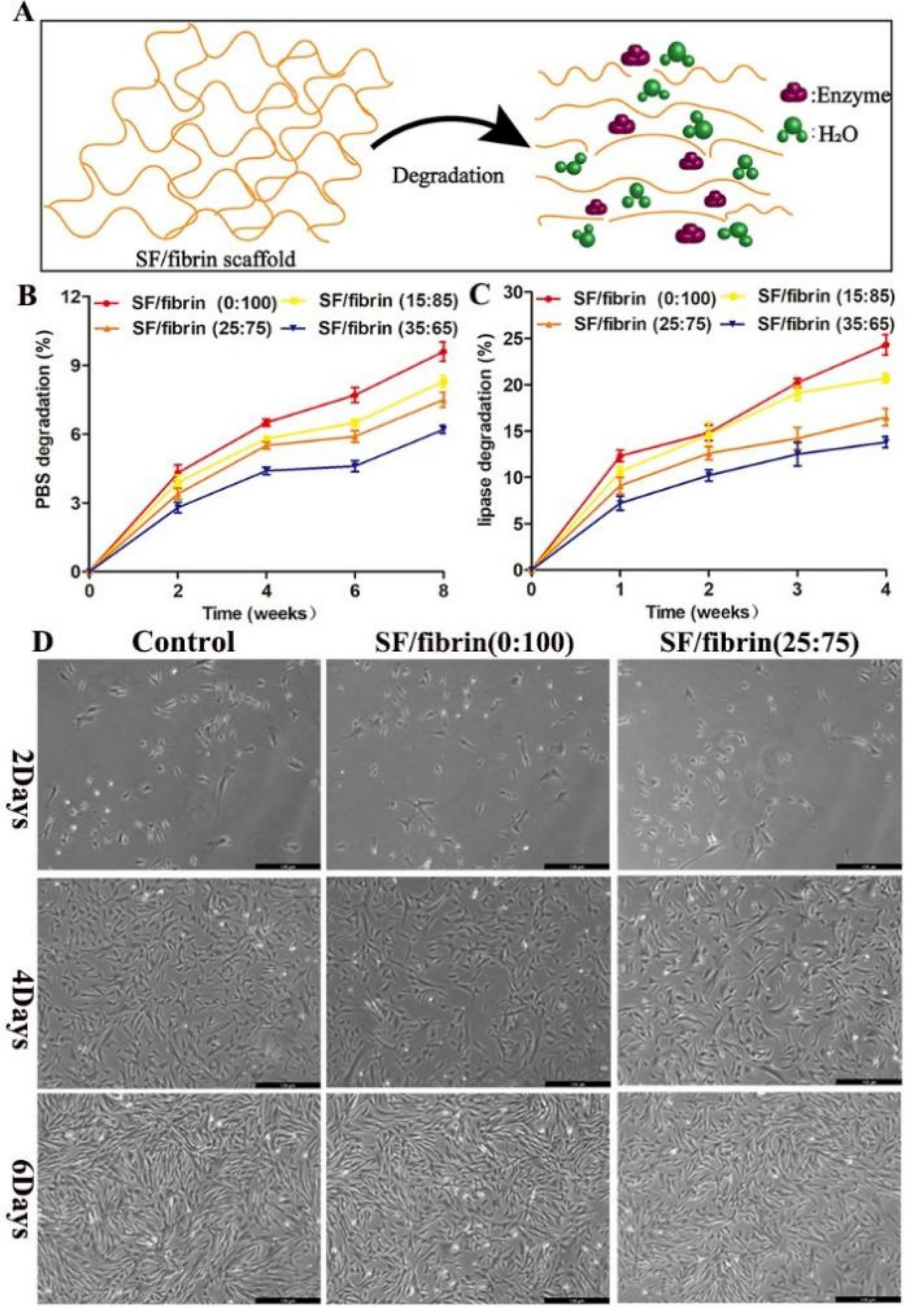
Schematic diagram of the degradation characterizations of SF/fibrin scaffold in vitro (**A**). Analysis of the PBS degradation (**B**) and lipase degradation (**C**) of the SF/fibrin electrospun materials with different mass ratios. The images of RADMSCs were cultured on different scaffold extracts at 2, 4 and 6 days respectively (**D**). Scale bar: 100 μm.

To determine the stability of the SF/fibrin scaffolds, we investigated its degradability in vitro. The degradation of scaffold materials includes water degradation and enzyme degradation which were shown in Fig. 4A. As the degradation time prolonged, the mass of different SF/fibrin scaffolds decreased. Notably, we found that the mass loss rate of the four scaffolds in PBS hydrolysis was much lower than that in lipase (Fig. 4B, C). We also observed slower degradation of the composite scaffolds that had an added SF component. The degradation rate of the SF/fibrin (0:100) electrospun scaffolds was higher than that of the other three composite scaffolds in PBS degradation (Fig. 4B). Moreover, the mass loss rate at lipase degradation of the SF/fibrin (15:85), SF/fibrin (25:75) and SF/fibrin (35:65) scaffolds were all lower than those of SF/fibrin (0:100) scaffolds after 4 weeks (Fig. 4C).

After 2, 4 and 6 days of co-culturing the different scaffold extracts and RADMSCs, we found that the cells grew in a typical spindle shape or fish school shape under the microscope. Concurrently, the morphology of these cells had not changed significantly. In addition, the number of cells in different sample groups were not statistically significant. The images of the three different groups showed that the corresponding cell amounts of cells increased gradually with the prolongation of the co-culturing time (Fig. 4D).

### Cell proliferation characteristics of the SF/fibrin scaffold

**Figure 5:**
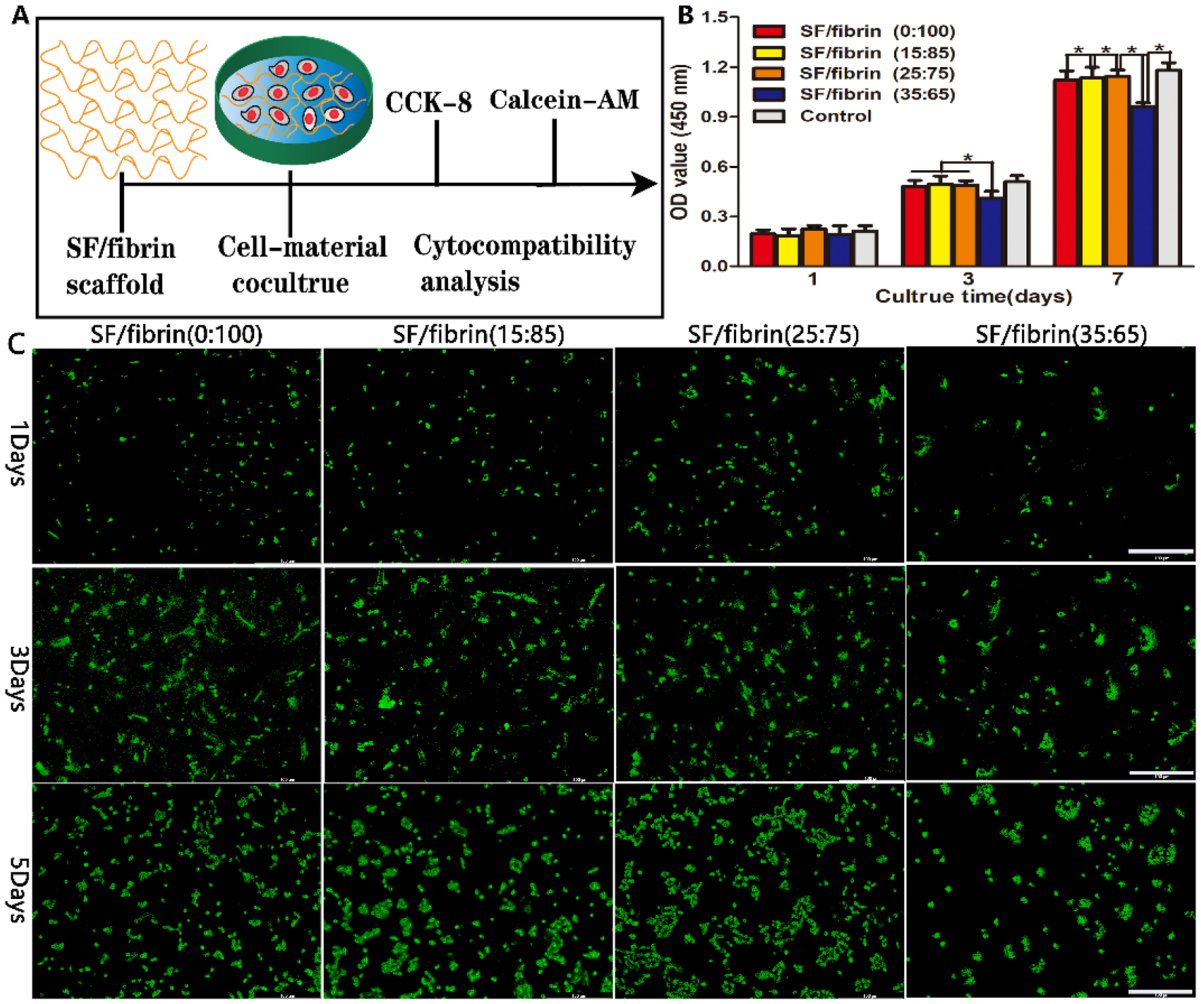
Proliferation behavior of RADMSCs on the four SF/fibrin electrospun scaffolds. The schematic diagram of the cytocompatibility test of scaffold materials in vitro (**A**). The optical density 450 values of the CCK-8 experiment was evaluated at 1, 3 and 7 days (**B**) (n = 3). Live (green) staining of the cells cultured on the four SF/fibrin blended scaffolds after different time points (**C**). Scale bar was 100μm.*P < 0.05.

To analyze the effect of the scaffold materials on the cell activity, we used RADMSCs as model cells, co-culturing them with the different SF/fibrin electrospun membranes (Fig. 5A). We assessed the proliferation behavior of the cells on different samples with CCK-8 kit assays. The results indicated that the number of cells on the different groups increased significantly over time. We observed no significant differences across the five groups after 1 day. However, we were surprised to observe that there was a significant statistical difference between the SF/fibrin (35:65) group, SF/fibrin (0:100) groups, SF/fibrin (15:85) groups and SF/fibrin (25:75) groups at 3 days of co-culture. Interestingly, the cell proliferation of the five groups was statistically significant at 7 days (Fig. 5B). After 1, 3, and 5 days of co-culture, we used a living cell staining test to assess the viability of the cells on different vascular materials (Fig. 5C). When this kit is used, live cells are transformed into fluorescent calcein by non-fluorescent Calcein AM in this kit, resulting in the green fluorescence. We successfully cultured the cells on different scaffold materials for 1, 3, and 5 days, respectively. Regardless of the day, the images showed that these cells on the random vascular scaffolds lacked any obvious orientation. The results clearly indicated that the number of living cells on SF/fibrin (15:85) and SF/fibrin (25:75) scaffolds was exceed that on the SF/fibrin (0:100) at different time points. However, the number of living cells on the SF/fibrin (35:65) scaffolds was much lower compared with the other groups.

### Analysis of cytoskeletal by fluorescence staining

**Figure 6:**
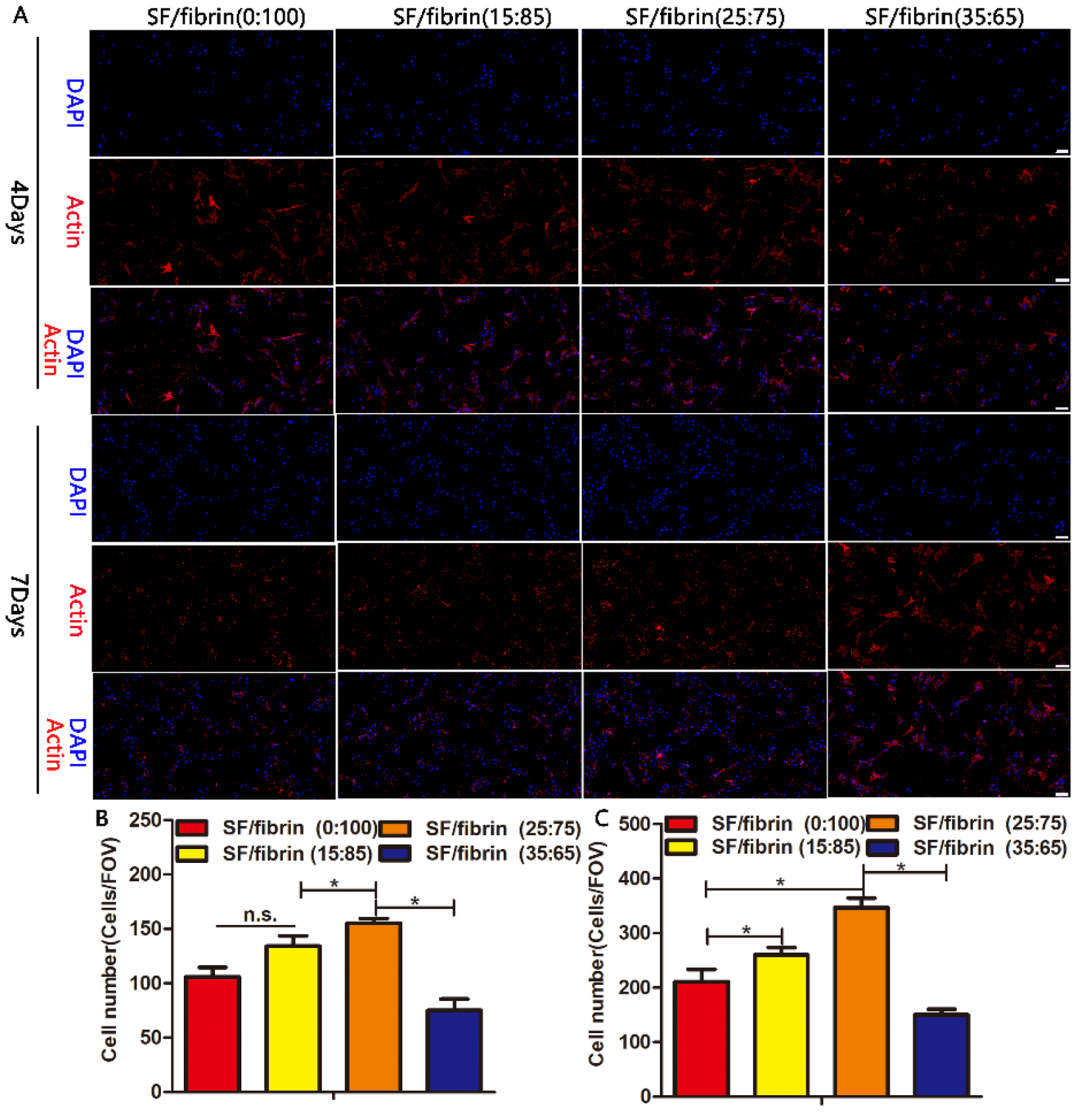
The adhesion of RADMSCs on different SF/fibrin electrospun membranes. The cytoskeleton (red) and DAPI (blue) staining images of the cells cultured on the scaffold material after 4 and 7 days, respectively (**A**). Scale bar = 100μm. Image J software was used to quantitatively evaluate the number of cells in the four groups after 4 days (**B**) and 7 days (**C**) of cultured (n = 3). *P < 0.05. n.s. represented no statistical significance.

After 4 days and 7 days of culturing, we evaluated adhesion and growth of the cells on the surface of the four different vascular scaffolds using actin (red) and DAPI (blue) staining assay. The cytoskeleton stained by actin antibody spread homogeneously along the direction of the MSCs on all different SF/fibrin electrospun membranes (Fig. 6A). Compared with the results after 4 days of cultured, the cell proliferation on the electrospun SF/fibrin scaffolds surface was extremely prosperous at 7 days. Surprisingly, the number of positive cells on the SF/fibrin (0:100), SF/fibrin (15:85) and SF/fibrin (25:75) scaffold was significantly greater than on the SF/fibrin (35:65) scaffold. At the same time, we found that the expression of actin on the surface of all four scaffold groups also increased with the prolong of culture time. To further verify this conclusion, these values were analyzed by Image J software. The statistical analysis confirmed that the number of cells on SF/fibrin (25:75) electrospun materials greatly exceeded that on the other groups after the 4 days and 7 days (Fig. 6B, C). Furthermore, the number of cells on the SF/fibrin (35:65) group was statistically different from the numbers for the other groups at the predetermined time point.

### Subcutaneous implantation of the vascular scaffold and degradation in vivo

**Figure 7:**
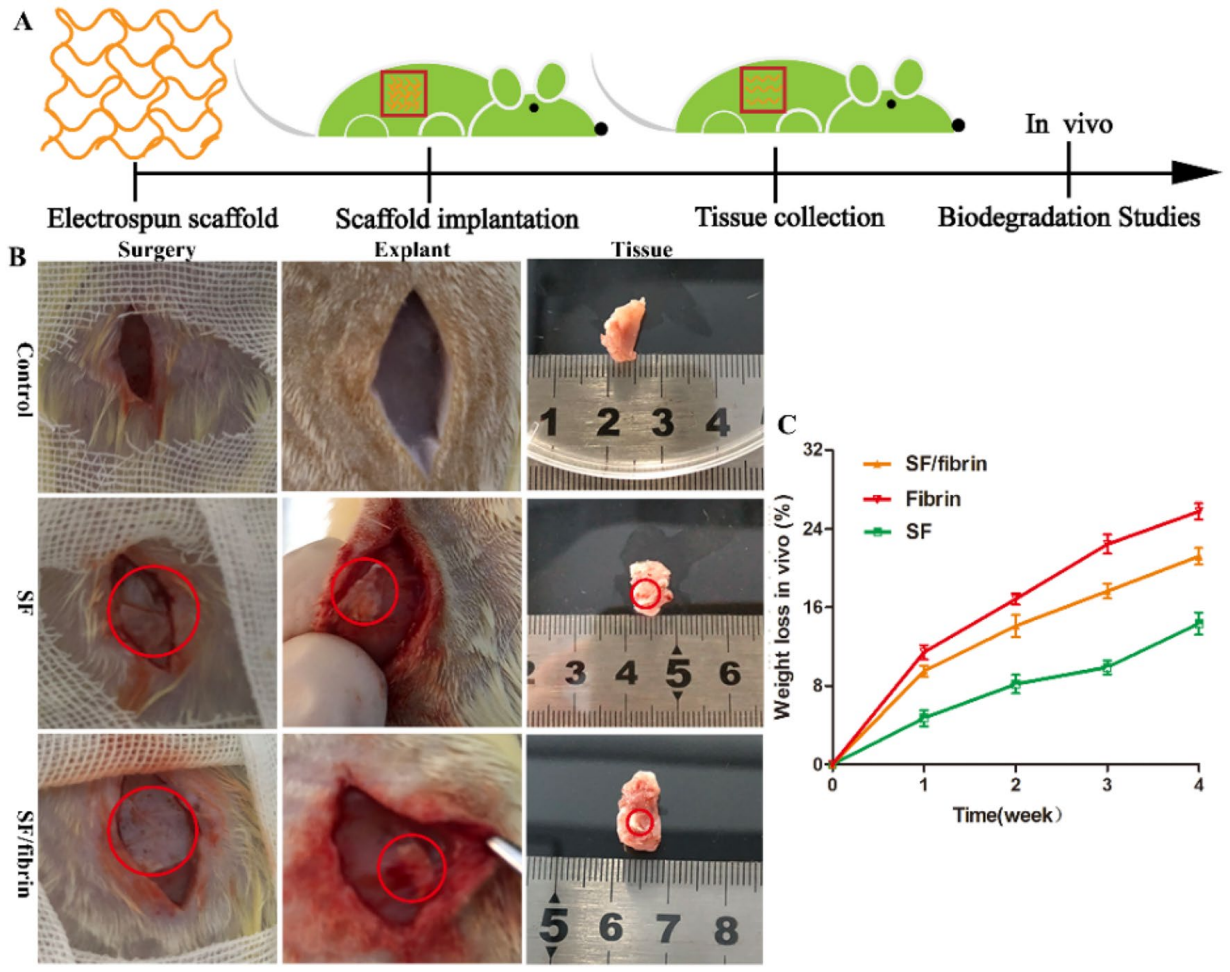
The schematic image of vascular scaffold degradation in vivo (**A**). Gross observation of the different vascular scaffolds after subcutaneous implantation for 2 weeks (**B**). The red circles indicated electrospun scaffold materials. The weight loss changes of three different scaffolds in vivo degradation (**C**).

We implanted the electrospun materials into animals to observe their degradation behavior in vivo (Fig. 7A). During the in vivo animal experiment, these SD rats showed no obvious signs of surgical incision infection or other adverse disease complications. Meanwhile, the sham surgery control group was established. Furthermore, we analyzed the morphological changes of electrospun scaffolds to investigate the in vivo degradation of different vascular materials after implantation. Two weeks after implantation, the surrounding tissues had wrapped completely around the scaffold materials. Moreover, we observed obvious changes in the appearance of the electrospun membranes. After completely removal of these scaffolds from the implantation sites, we observed that the size of SF/fibrin (25:75) scaffold material to be smaller than that of SF materials, suggerting some degree of degradation (Fig. 7B). Meanwhile, we also analyzed that the degradation rate of the SF vascular scaffolds was lower than that of SF/fibrin (25:75) scaffolds while the degradation rate of the pure fibrin scaffolds was higher than their group. Four weeks after implantation, the degradation rate of SF, SF/fibrin (25:75) and fibrin scaffolds were 14.34%, 21.20% and 25.76%, respectively (Fig. 7C).

### Investigation of host histology in vivo

**Figure 8:**
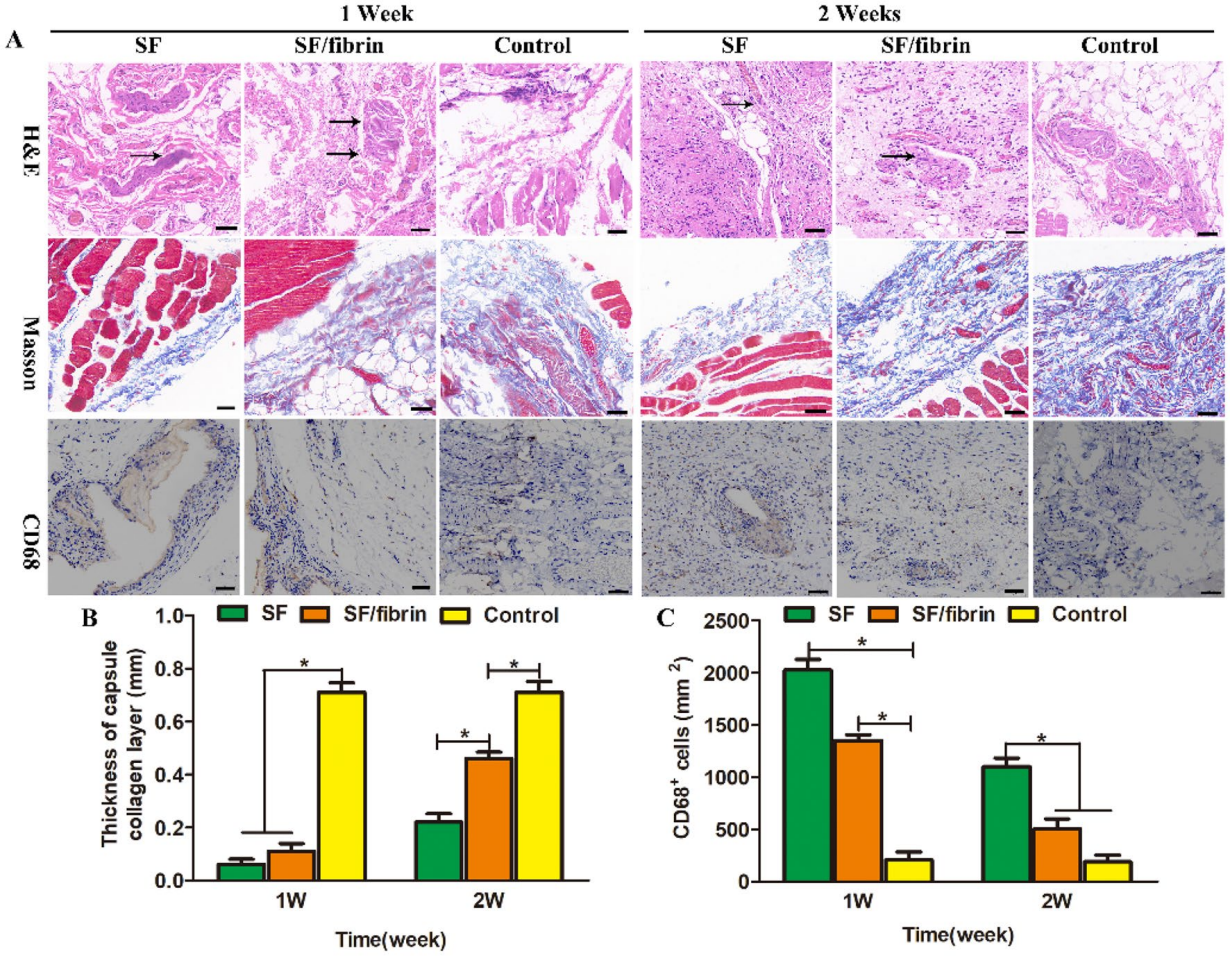
Histological images of SF group, SF/fibrin (25:75) and control group after subcutaneous implantation for 1 week or 2 weeks (**A**). The scale bar of H&E staining, Masson staining and CD68 staining images indicated 50 μm, respectively. The black arrowed marked the scaffold. The quantitative evaluation of thickness of capsule collagen layer (**B**) and CD68 positive cells (**C**) in different groups (n = 3). *P < 0.05 in (**B**, **C**).

The results confirmed that compared with the control group, the SF scaffold and SF/fibrin (25:75) scaffold more obviously induced greater infiltration of inflammatory cells into the host tissue after 1 week of implantation. At 2 weeks after implantation, the degree of inflammatory reaction in all of the groups decreased significantly compared with the reaction before implantation. At the same time, the inflammatory reaction in SF/fibrin (25:75) group was milder than that in SF group (Fig. 8A). To evaluate the content of collagen deposition in blue, we used Masson’s trichrome staining. Images of the Masson’s staining showed considerable collagen deposition in the different groups, and then the SF scaffold induced more sparser fibrous capsules than the SF/fibrin (25:75) scaffold at 1 and 2 weeks. These results confirmed that the SF/fibrin (25:75) scaffold developed an abundant and well-organized collagen structure while the SF exhibited some the fibrous electrospun scaffold structure and a small amount of newly collagen formation. Moreover, the results of the quantitative analysis confirmed that collagen layer in SF/fibrin (25:75) vascular scaffold was significantly thicker than in the SF vascular scaffold, and thinner than that in the control group (Fig. 8B). The CD68 antibody markers served to analyze the inflammatory response of the three different samples. After 1 week, the immunohistochemistry images of CD68 showed that the SF scaffolds induced a significantly stronger immune response than that in the SF/fibrin (25:75) scaffold. Interestingly, we were surprised to observe that the expression level of CD68 in all groups decreased with the prolongation of the implantation time. What’s more, the quantitative calculation results showed that the expression of CD68 positive cells in SF group was significantly higher than that in the other two groups. Furthermore, we observed no significant difference in the number of CD68 cells between the SF/fibrin and the control group at 2 weeks (Fig. 8C).

## Discussion

Many researchers have shown that the ideal preparation characteristics of small-diameter tissue engineering vascular scaffolds are stable mechanical strength, biodegradability, excellent biocompatibility, minimal immune rejection and easy operation^19^. Meanwhile, effective vascular scaffolds should also facilitate regeneration and vascular reconstruction in vivo^20^. Moreover, many scientists have successfully used SF and fibrin as raw materials to construct artificial vascular scaffolds that have been widely applied to small-caliber tissue engineering blood vessels^21,22^. In the research described here we used electrospinning technology to fabricate SF/fibrin composite materials with different mass ratios, and then systematically analyzed their mechanical properties, hemocompatibility, degradability, cytocompatibility, and the biocompatibility of the vascular scaffolds made from them. We found that the SF/fibrin fiber scaffolds were uniformly distributed, with a smooth surface and suitable porosity (Fig. 1C1–C4). Above all, the results showed that the fiber diameter of the electrospun vascular materials decreased gradually with the increase in the SF content of SF/fibrin scaffolds. This result is mainly due to the density of SF/fibrin spinning solution increased after the addition of SF, thereby enhancing its conductivity, resulting in a relatively small fiber diameter of its composite scaffold (Fig. 1D1–D4).

Generally speaking, the hydrophilicity of a biomaterial surface seriously affects the biocompatibility of the vascular scaffold. Researchers have demonstrated that the surface of suitable hydrophilic substances was promotes cell growth, proliferation, and adhesion^23,24^. From Fig. 2A, we were surprised to observe that the water contact angle of the vascular scaffold also increased significantly with the increase in the SF content of the SF/fibrin scaffold. These results may be explained by the increase in the β-sheet structure of the SF molecules, which prevented water molecules from infiltrating into materials, and then led to the decrease of hydrophilicity. Therefore, the hydrophilic property of the composite scaffold was greatly enhanced by the addition of fibrin material with good hydrophilicity.

Furthermore, the mechanical properties serve as an important reference index for tissue engineering vascular scaffolds. Artificial vascular scaffolds with mechanical properties similar to those of native vessels can not only withstand the force of the blood flow, but also maintain the normal shape of the vascular lumen^25^. An important evaluation factor, then, is burst strength, which reflects the resistance of the artificial scaffold to blood pressure in vivo. In this study, the burst pressure value of SF/fibrin (15:85), SF/fibrin (25:75) and SF/fibrin (35:65) electrospun scaffolds were higher than those of the human native blood vessels (1500 mmHg) and similar to those of the saphenous veins (1680–2273 mmHg)^26,27^. These results further confirmed that the SF/fibrin vascular scaffolds met the requirements for tissue engineering blood vessels. The suture strength is the maximum suture tension between the scaffold material and the vascular anastomosis. From Fig. 2C, the suture strength values of the SF/fibrin (25:75) and SF/fibrin (35:65) vascular scaffolds were 0.43 N and 0.55 N, respectively, indicating that they were closed to the requirements for artificial blood vessels (0.5 N)^28^. Additionally, the results also showed that the tensile strength value and the elongation at break value of the four SF/fibrin vascular scaffolds increased obviously with increased SF content. These results confirmed that the addition of SF can significantly enhance the mechanical properties of SF/fibrin electrospun scaffolds. Nevertheless, the elastic modulus values of different SF/fibrin vascular scaffolds showed that the addition of SF could improve their elastic deformation capacity. More importantly, related studies have demonstrated that the β-sheet content of SF is directly proportional to the correlation among the mechanical properties^29^. Hence, our results showed that the mechanical strength of the vascular scaffolds may be closely related to the makeup of the SF material, the degree of crystallization, and β-sheet formation.

Various physical and chemical forces may the rupture and dissolve red blood cells, and the release of hemoglobin, which is referred to as hemolysis^30^. The results shown in Fig. 3B indicated that none of our SF/fibrin electrospun scaffolds exceeded 2%, confirming that the vascular scaffold is reliable and safe for clinical application. The adherence of platelets to collagen tissue and the surfaces of foreign bodies (platelet adhesion) contributes significantly to the promotion of thrombotic transduction by vascular materials^31^. This outcome that may be due to the successful mixing of the fibrin material with superior hydrophilicity and the SF material with a rich negative charge, thereby greatly reducing the number of platelet adhesion to SF/fibrin vascular scaffolds. Furthermore, the red blood cells on different nanomaterials exhibited good morphology. Our analysis showed from the results that the hydrophilic SF/fibrin scaffolds were highly compatible with red blood cells^32^. The PRT was evaluated to determine the time at which coagulation occurred in the electrospun scaffold after the addition of calcium ions^33^. We found no statistically significant difference in the PRT values of four different SF/fibrin electrospun scaffolds. These results demonstrated that the blood compatibility of the scaffold did not decrease with the increased of SF content. Recently, APTT and TT experiments have been widely used to analyze the antithrombogenicity function of biomaterials in vitro. Our results further showed that changing the SF content had no effect on the anticoagulation property of the SF/fibrin composite scaffold.

During the remodeling process of artificial blood vessels, a balance is needed provided between the degradation rate of ideal biomaterials and the regeneration of tissue cells in vivo^34^. The results shown in Fig. 4B indicated that the degradation rate of the four SF/fibrin scaffolds in PBS increased as the fibrin content decreased. Specifically, the mass loss of the SF/fibrin (0:100) scaffold exceeded that of other groups after 8 weeks of degradation. Li et al.^35^ had successfully confirmed that fibrin gel is an ideal biodegradable scaffold, and the material adhering to native tissues after its degradation so as to promote migration, attachment and proliferation. In the meantime, we found that the trend of results in the enzymatic degradation was comparable to that in PBS (Fig. 4C). This result may be due to the corresponding biochemical reaction that occurs when the enzyme enters the internal active site of the scaffold, destroying the molecular structure of the composite scaffold^36^. Accordingly, fibrin showed excellent degradation performance, and we designed related in vitro experiments to analyze the biocompatibility of SF/fibrin electrospun scaffolds. From the results, we observed that these cells in the SF/fibrin group exhibited superior cell migration, attachment, and viability compared with those in the control group after 2, 4, and 6 days of cultured (Fig. 4D). These results may be attributable to the result of the complementary roles of SF and fibrin in the cellular response of MSCs.

Recently, numerous scientific researchers have prepared vascular materials through electrospinning that mimic the structure and functionality of the extracellular matrix and have the potential to improve the growth and proliferation behavior of cells significantly^37^. For example, Karkan et al.^38^ analyzed the compatibility between PU/PCL nanofibers with different ratios and HUVECS and found that the cells on the vascular materials exhibited good proliferation behavior. In the CCK-8 experimental consequences, we analyzed that the cell counts of the five groups increased over time (Fig. 5B). After 3 and 7 days of co-culture, the cell proliferation on the SF/fibrin (0:100), SF/fibrin (15:85) and SF/fibrin (25:75) scaffolds was significantly better than that on SF/fibrin (35:65), but still lower than the control group. This results fully confirmed that the SF/fibrin (25:75) electrospun scaffolds can effectively improve the cell proliferation behavior on the scaffold material with relatively low toxicity. To better verify this phenomenon, we evaluated it by living cell fluorescence experiment. By fluorescence staining microscope, we observed that these cells on four SF/fibrin scaffolds groups showed good growth and adhesion ability. Further, compared with the other three groups, the number of live cells on the SF/fibrin (35:65) vascular scaffolds in that group was the lowest (Fig. 5C). Additional results further indicated that the SF/fibrin (25:75) vascular scaffold exhibited excellent cytocompatibility which could be considered as one of the ideal tissues engineering vascular scaffold.

To evaluate further the activity of cells on the different vascular scaffolds at specific time points, we carried out a series of related immunofluorescence experiments. Many researchers have reported that the chemical and compositional changes in pure SF vascular scaffolds prepared by electrospinning improved cell viability. Furthermore, as medical suture material of SF had displayed good biocompatibility with many types of cells and could promoted the growth of vascular cells^39^. In our research for this study, we were surprised to observe that the number of cells in the SF/fibrin (25:75) group was greater than that in the SF/fibrin (15:85) and SF/fibrin (35:75) groups. Meanwhile, the results shown in Fig. 6 indicated that the SF/fibrin (15:85) group was slightly more adhesive than the SF/fibrin (0:100) group. This result may be due to the high surface to volume ratio and nanofibrous biomimetic architecture of the vascular scaffolds prepared by electrospinning technology, which are more conducive to the attachment and growth of the cells on the scaffold materials. We also analyzed the proliferation morphology of different electrospun scaffolds under a fluorescence microscope. All our results indicated that SF/fibrin composite vascular materials exhibited superior biocompatibility.

A crucial consideration in the application of vascular scaffolds is that the in vivo degradation rate should match the tissue regeneration rate^40^. The main influences on the degradation of biomaterials are the microenvironment, the structure and morphology of the biomaterials, bioactive substances such as enzymes, and macrophages. Wang et al.^41^ prepared three-dimensional porous scaffolds from regenerated silk fibroin and evaluated their degradation behavior in rats at 8 weeks and 1 year, respectively. We concluded that the multiple factors such as its preparation method, structural composition and the host’s immune response affected the degradation in vivo of the scaffold, thus showing further that the in vivo degradation of the scaffolds was predictable and controllable. We found that the degradation rate of the SF/fibrin vascular scaffolds in vivo to be higher than that of the SF scaffolds. At the same time, the sizes of the electrospun samples decreased as the implantation time increased, and the scaffolds adhered to the surrounding tissues (Fig. 7B). Our analysis indicated that SF with a content of silk II structure may effectively reduce the degradation rate of the biomaterial sufficiently. Additionally, unlike the fibrin scaffold, which had a higher degradation rate and the SF scaffold, which had a lower degradation rate, the SF/fibrin was able to maintain a reasonable degradation rate in vivo that greatly enhances tissue regeneration and remodeling.

The implantation of electrospun scaffold materials into SD rats can commonly leads to inflammatory body reactions and adherence to the host tissue to promote fibrous encapsulation^42^. We removed the surrounding explants for histological evaluation after 1 and 2 weeks to confirm that the tissues surrounding the SF and SF/fibrin scaffolds demonstrated a significant inflammatory cell response. Meanwhile, the expression of macrophages in different samples was further evaluated by CD68 staining. The number of CD68 cells on SF/fibrin scaffold was less than that on SF scaffold, and then the expression level of CD68 on all samples changed with the implantation time (Fig. 8). These results confirmed that the SF/fibrin scaffold can enhance the cellular infiltration and change in phenotype of macrophages. Collagen, the deposition of which is a key step in vascular regeneration, is the main component of the constructed extracellular matrix. Our results indicated that the SF/fibrin scaffolds had thicker collagen than the SF scaffolds at different times, but were slightly thinner than the control group. Thus, the SF/fibrin vascular scaffold displayed good tissue regeneration ability and has the potential to serve as an ideal artificial blood vessel scaffold.

## Conclusion

In this study, we used electrospinning equipment to fabricate SF/fibrin vascular scaffolds. We found that the fiber diameter of the pure fibrin scaffold to be significantly larger than that of the SF/fibrin scaffolds with other mass ratios. These results indicated that the mechanical performances of the SF/fibrin vascular scaffolds increased as the SF content increased, with significantly effects on the surface hydrophilicity and degradation rate of vascular scaffolds. Nevertheless, the SF content of SF/fibrin scaffold was 35%, and the cell compatibility was obviously impaired. Therefore, SF/fibrin (25:75) electrospun scaffold demonstrated balanced mechanical strength, excellent hemocompatibility, biodegradability and cytocompatibility. Importantly, a series of in vivo experiments showed that the degradation rate of the SF/fibrin scaffold was higher than that of the SF scaffold. Further, the expression of collagen in the SF/fibrin (25:75) vascular scaffolds was higher than that in pure SF scaffolds, but their macrophage content was relatively low. We confirmed that SF/fibrin scaffold was considered as a candidate composite material for small diameter tissue engineering vascular scaffolds.

## Data Availability

All data generated or analyzed during this study are included in this published article.
